# Essential Role of CRIM1 on Endometrial Receptivity in Goat

**DOI:** 10.3390/ijms22105323

**Published:** 2021-05-18

**Authors:** Diqi Yang, Ai Liu, Yanyan Zhang, Sha Nan, Ruiling Yin, Qianghui Lei, Hongmei Zhu, Jianguo Chen, Li Han, Mingxing Ding, Yi Ding

**Affiliations:** Department of Clinical Veterinary Medicine, College of Veterinary Medicine, Huazhong Agricultural University, Wuhan 430070, China; diqiyang@mail.hzau.edu.cn (D.Y.); liuai@webmail.hzau.edu.cn (A.L.); hzaudongyizyy@163.com (Y.Z.); nansha@webmail.hzau.edu.cn (S.N.); yinruiling0925@163.com (R.Y.); leiqqqqqh@163.com (Q.L.); zhuhongmei@mail.hzau.edu.cn (H.Z.); chenjg@mail.hzau.edu.cn (J.C.); hanli209@mail.hzau.edu.cn (L.H.)

**Keywords:** CRIM1, autophagy, hormones, endometrial receptivity, goats

## Abstract

In domestic ruminants, endometrial receptivity is related to successful pregnancy and economic efficiency. Despite several molecules having been reported in the past regarding endometrial receptivity regulation, much regarding the mechanism of endometrial receptivity regulation remains unknown due to the complex nature of the trait. In this work, we demonstrated that the cysteine-rich transmembrane bone morphogenetic protein (BMP) regulator 1 (CRIM1) served as a novel regulator in the regulation of goat endometrial receptivity in vitro. Our results showed that hormones and IFN-τ increased the expression of CRIM1 in goat endometrial epithelial cells (EECs). Knockdown of CRIM1 via specific shRNA hindered cell proliferation, cell adhesion and prostaglandins (PGs) secretion and thus derailed normal endometrial receptivity. We further confirmed that receptivity defect phenotypes due to CRIM1 interference were restored by ATG7 overexpression in EECs while a loss of ATG7 further impaired receptivity phenotypes. Moreover, our results showed that changing the expression of ATG7 affected the reactive oxygen species (ROS) production. Moreover, mR-143-5p was shown to be a potential upstream factor of CRIM1-regulated endometrial receptivity in EECs. Overall, these results suggest that CRIM1, as the downstream target of miR-143-5p, has effects on ATG7-dependent autophagy, regulating cell proliferation, cell adhesion and PG secretion, and provides a new target for the diagnosis and treatment of early pregnancy failure and for improving the success rates of artificial reproduction.

## 1. Introduction

In domestic ruminants, early pregnancy failure is one of the main reasons why pregnancy cannot be established, and suboptimal endometrial receptivity is responsible for two-thirds of implantation failures [1,2]. During the “window of receptivity”, the endometrial epithelium undergoes a dynamic change that is stimulated and maintained by maternally derived progesterone (P_4_), estradiol (E_2_) and conceptus-derived interferon-tau (IFN-τ) [3]. Hormones and IFN-τ induce the complementary expression of regulators, which is critical for anchoring the implanting blastocyst to the apical surface of the luminal epithelium [4]. Recent evidence suggests that Homeobox proteins A10 and A11 (HOXA10, HOXA11) are important for endometrial receptivity, since their deletion induces abnormal uterine stromal and glandular function [5,6]. Integrin subunit beta 1 (ITGB1), integrin subunit beta 3 (ITGB3) and integrin subunit beta 5 (ITGB5) are members of integrins, which interact with the extracellular matrix (ECM) to establish endometrial receptivity and embryo implantation [7]. Secreted phosphoprotein 1 (SPP1) is an ECM protein that is localized to the uterine luminal epithelium during implantation in sheep and binds integrin receptors to promote assembly of focal adhesion [8]. According to previous studies, these molecules are well recognized as markers of endometrial receptivity [9,10,11].

In the past decade, omics—which refers to the application of high-throughput techniques—have been applied to explore endometrial physiology, and it has been found that a large number of genes are involved in the regulation of endometrial receptivity [12,13,14]. One of the studies using transcriptome analyses reported that a higher level of cysteine-rich transmembrane BMP regulator 1 (CRIM1) was observed in the receptive endometrium (RE) than the pre-receptive endometrium (PE) by strand-specific Ribo-Zero RNA-Seq and quantitative-PCR in goats [15]. CRIM1 is a type I transmembrane protein, with an N-terminal insulin-like growth factor-binding protein motif (IGFBP) and six cysteine-rich von Willebrand factor C (vWC) repeats located in the extracellular domain [16]. It has been suggested that CRIM1 is an indispensable factor in cell polarity, proliferation, adhesion and angiogenesis [16,17,18]. The above-mentioned physiological processes are necessary to establish adequate maternal–embryonic crosstalk. These results have led to the speculation that CRIM1 may play a central role in endometrial receptivity in goat.

Autophagy is a highly conserved biological behavior for the maintenance of cellular homestasis and plays an indispensable role in physiological and pathophysiological processes related to reproduction in mammals [19]. The mechanistic/mammalian target of rapamycin (mTOR), autophagy related 7 (ATG7), microtubule-associated protein 1 light chain 3 (LC3), sequestosome 1 (SQSTM1), transcription factor EB (TFEB) and lysosomal-associated membrane protein 1 (LAMP1) are considered to be essential molecules for the induction of autophagy. It is known that mTOR is phosphorylated at Ser2448 and negatively regulates catabolic processes such as autophagy [20]. ATG7 is necessary for the conjugation of ATG12 to ATG5 as an E1-like enzyme and then mediates the ATG5–ATG12 ubiquitin-like conjugation pathways [21]. During autophagy, a cytosolic form of LC3-I is cleaved and then conjugated to phosphatidylethanolamine to form the LC3-II, which is a marker of autophagosomes [22]. Then, SQSTM1 (also known as P62) interacts with LC3 to recognize ubiquitylated proteins targeted for degradation [23]. Lotfi et al. have reported that TFEB serves as a master regulator of lysosomal function by governing lysosomal biogenesis and autophagy, which is induced by its translocation from cytoplasm to nucleus under autophagic stimuli [24]. LAMP1 is involved in autophagy by mediating the end-stage degradation of autophagy [25]. Moreover, autophagy is characterized by the accumulation of autophagosomes in the cytoplasm [23]. Previous studies have reported that autophagy can be triggered in the endometrial cell cycle during the late secretory phase [26]. Recently, studies have found that autophagy gene ATG16L1 is a potential mediator of human endometrial decidualization [19]. Accumulating evidence indicates that autophagy shows a close relationship with the cell proliferation, angiogenesis, cell apoptosis, prostaglandin secretion and cell attachment of endometrial epithelial cells [27,28,29]. In particular, three recent studies have reported that autophagy appears to be the cruical pathway of endometrial regulation during early pregnancy in goat [30,31,32].

MicroRNAs (miRNAs), which serve as post-transcriptional regulatory molecules, are essential for regulating gene expression by binding to 3′ untranslated regions (3′UTRs) to degrade or repress the translation of mRNA [33]. To date, plenty of studies have shown the association between miRNA and physiology, as well as in endometrial receptivity. An et al identified miR-449a could promote pinopode formation during embryo implantation and enhance endometrial receptivity [34].MiR-26a might play an important role in the regulation of endometrial cell proliferation and the development of endometrial receptivity [35]. MiR-143-5p is highly conserved in vertebrates and has been found to be differentially expressed in the RE and the PE in goats [36]. Tian et al. reported that miR-143 may be involved in the process of blastocyst implantation by regulating Lifr in rat [37]. However, miR-143 may play a crucial role in blastocyst implantation, with a handful of recent studies identifying the potential mechanism of miR-143-5p in the regulation of goat endometrial receptivity.

Despite the in-depth understanding of the processes associated with the development of various tissues and CRIM1, little progress has been achieved regarding its role in endometrial receptivity. We therefore hypothesized that CRIM1 may serve as a novel regulator of endometrial receptivity in goat. To examine this hypothesis, we measured the effectiveness of CRIM1 on cell proliferation, cell adhesion and PG secretion. Our results showed that CRIM1 interference hampered cell proliferation, cell adhesion and PG secretion and thus derailed normal endometrial receptivity in goat. Mechanistically, we demonstrated that CRIM1, as the downstream target of miR-143-5p, has effects on ATG7-dependent autophagy, regulating receptivity phenotypes. Moreover, the formation of reactive oxygen species (ROS) may be a crucial signaling molecule for the development of endometrial receptivity.

## 2. Results

### 2.1. Hormones and IFN-τ Treatment Increased CRIM1 and Activated Autophagy

**Figure 1:**
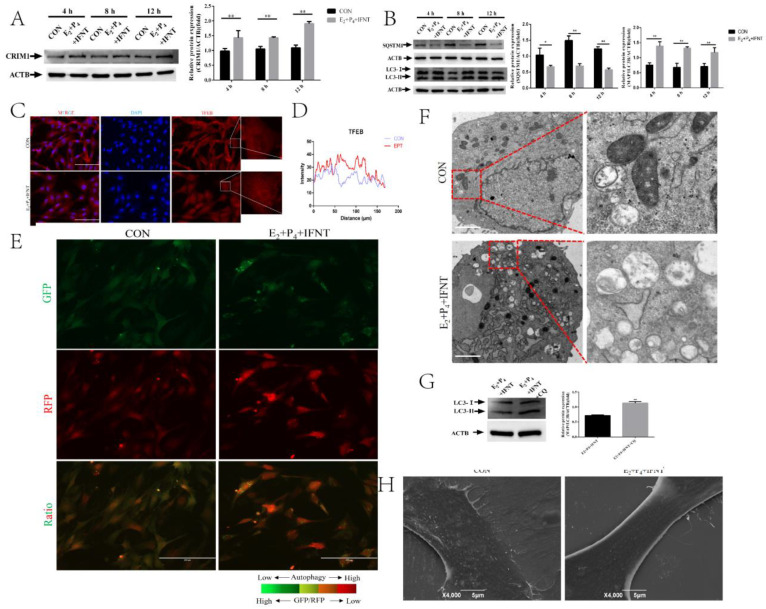
Hormones and IFN-τ treatment increased CRIM1 and activated autophagy. (**A**,**B**) After being treated with or without P_4_, E_2_ and IFN-τ, total proteins from EECs were subjected to Western blotting. (**C**,**D**) Fluorescence microscopy images of TFEB expression in EECs and histograms of fluorescence intensity. Representative images of three independent experiments are shown. Scale bar = 100 μm. (**E**) The GFP/RFP fluorescence ratio images of EECs with or without P_4_, E_2_ and IFN-τ treatment are shown. (**F**) EECs were treated with or without P_4_, E_2_ and IFN-τ for 12 h. The EECs were fixed and processed for TEM analysis. Scale bar = 2 μm. (**G**) After being pre-treated with or without CQ, total proteins from EECs were subjected to Western blotting. (**H**) Scanning electron microscopy (SEM) of EECs, which were treated with or without P_4_ and E_2_ followed by IFNT incubation for 12 h. The data are presented as the means ± SEM of three independent experiments. * Significant difference (*p* < 0.05) compared with other groups; ** Significant difference (*p* < 0.01) compared with other groups.

P_4_, E_2_ and IFN-τ are considered to be essential for endometrial receptivity in ruminants [38]. Our first set of questions aimed to elucidate whether the expression of CRIM1 is regulated by P_4_, E_2_ and IFN-τ treatment. The Western blot results revealed that P_4_, E_2_ and IFN-τ treatment significantly upregulated CRIM1 expression in endometrial epithelial cells (EECs) compared to those in the control (CON) groups at 4 h, 8 h and 12 h (Figure 1A). Previous studies have indicated that the expression of autophagosome-associated LC3-II, a marker of autophagosomes, was upregulated under P_4_, E_2_ and IFN-τ treatment [31]. We found that P_4_, E_2_ and IFN-τ treatment increased the activation of the autophagy flux of LC3-II expression and induced SQSTM1 degradation in EECs (Figure 1B). Immunofluorescence results showed that P_4_, E_2_ and IFN-τ drove the nuclear translocation of TFEB, and a significant increase of TFEB nuclear expression was found in the P_4_, E_2_ and IFN-τ group EECs (Figure 1C,D). According to Kaizuka’s description [39], we constructed the autophagy probe GFP-LC3-RFP, which monitored autophagic flux by calculating the GFP/RFP ratio. As shown in Figure 1E, the GFP/RFP ratio was decreased under P_4_, E_2_ and IFN-τ treatment. Next, transmission electron microscopy (TEM) was employed to determine the autophagosome levels. Structural analysis via TEM allowed for the visualization of autophagy in the EECs treated by P_4_, E_2_ and IFN-τ, characterized by the massive accumulation of autophagosomes in the cytoplasm (Figure 1F). To further confirm these findings, chloroquine (CQ) was used to block autophagosome fusion with lysosomes. As shown in Figure 1G, the CQ pre-treatment group exhibited an accumulation of LC3-II, indicating that the P_4_, E_2_ and IFN-τ treatment activated autophagic flux. Moreover, we found the CON group had distributed microvilli on the cell surface, whereas the surface of P_4_+E_2_+IFN-τ group cells was almost devoid of microvilli (Figure 1H).

### 2.2. Knockdown of CRIM1 Inhibited Autophagy

**Figure 2:**
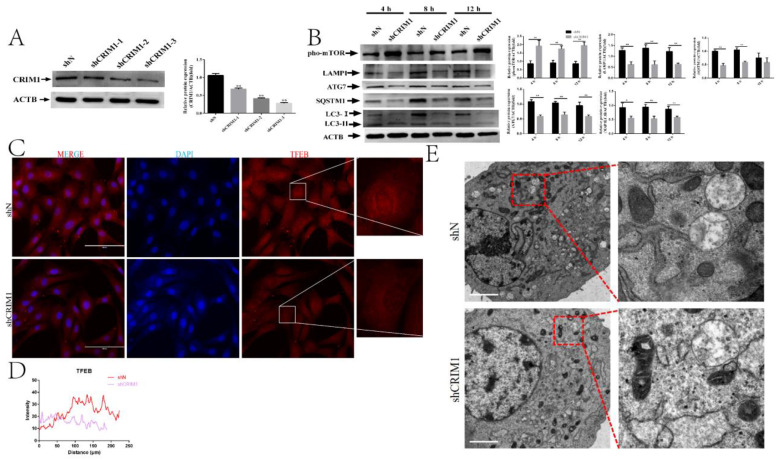
Knockdown of CRIM1-inhibited autophagy under P_4_, E_2_ and IFN-τ treatment. (**A**) EECs were infected with a lentivirus specific for CRIM1 and a negative lentivirus (shN) for 48 h. Western blot analysis of CRIM1 levels in EECs. (**B**) After being treated with P_4_, E_2_ and IFN-τ, total proteins from EECs were subjected to Western blotting. (**C**,**D**) Fluorescence microscopy images of TFEB expression in EECs and histograms of fluorescence intensity. Representative images of three independent experiments are shown. Scale bar = 100 μm. (**E**) EECs were treated with P_4_, E_2_ and IFN-τ for 12 h. The EECs were fixed and processed for TEM analysis. Scale bar = 2 μm. The data are presented as the means ± SEM of three independent experiments. * Significant difference (*p* < 0.05) compared with other groups; ** Significant difference (*p* < 0.01) compared with other groups.

To determine whether CRIM1 protein is required for hormone-induced autophagy, we measured the effects of shRNA-mediated CRIM1 depletion on P_4_, E_2_ and IFN-τ treatment-induced autophagy in EECs. We first constructed lentiviral shRNA vectors targeting the goat CRIM1 gene and infected EECs with the lentivirus. As shown in Figure 2A, the shCRIM1-3 group had the greatest downregulatory effect on the CRIM1 protein level. Therefore, shCRIM1-3 was used for subsequent studies. A Western blot was adopted to analyze the expression of pho-mTOR, ATG7, LC3-II, SQSTM1 and LAMP1 in the shN and shCRIM1 groups under P_4_, E_2_ and IFN-τ treatments at different time points. It can be seen from the data in Figure 2B that the EECs infected with CRIM1 shRNA showed an elevation in pho-mTOR expression and reductions in ATG7, LC3-II, SQSTM1 and LAMP1 levels. To clarify the role of CRIM1 on autophagy, we examined the subcellular localization of TFEB. Figure 2C,D show [40] that the quantification of nuclear TFEB expression in the shCRIM1 EECs was significantly decreased compared to the shN EECs. Furthermore, the TEM observation demonstrated that the number of autophagosomes was lower in the shCRIM1 EECs under P_4_, E_2_ and IFN-τ treatment (Figure 2E).

### 2.3. Knockdown of CRIM1 Jeopardized Endometrial Receptivity

**Figure 3:**
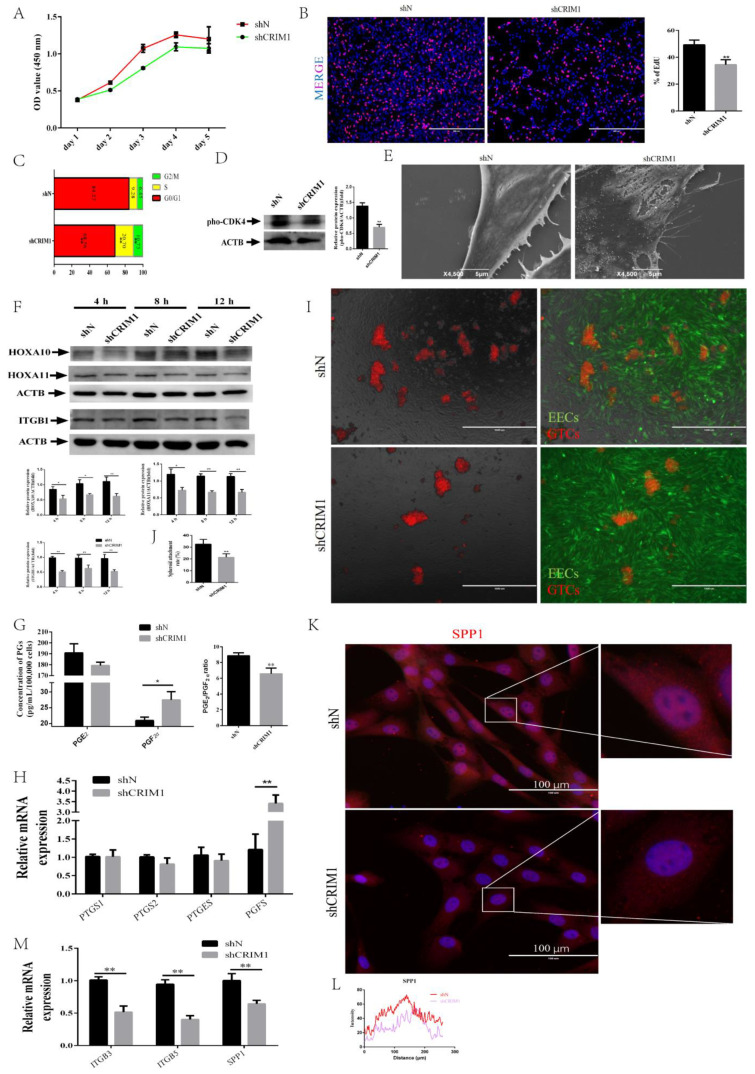
Knockdown of CRIM1 jeopardized endometrial receptivity. EECs were infected with a lentivirus specific for CRIM1 and shN for 48 h under P_4_, E_2_ and IFN-τ treatment. (**A**) Growth curves of the shN and shCRIM1 groups were determined by the CCK-8 method. (**B**) The shN and shCRIM1 groups were collected to measure DNA synthesis by EdU proliferation assay. (**C**) The shN and shCRIM1 groups were collected to measure the cell cycle distributions by flow cytometry. (**D**) Western blot analysis of pho-CDK4 expression in the shN and shCRIM1 groups. (**E**) The shN and shCRIM1 groups were treated with P_4_, E_2_ and IFN-τ for 12 h. The EECs were fixed and processed for SEM analysis. (**F**) After being treated with P_4_, E_2_ and IFN-τ, total proteins from the shN and shCRIM1 groups were subjected to Western blotting. (**G**) The secretion levels of PGE_2_ and PGF_2α_ were measured in EECs using an ELISA kit. (**H**) The rate-limiting enzymes of synthesized PGs were measured by real-time quantitative PCR. (**I**,**J**) GTCs spheroids were prepared, and the spheroid attachment rate was measured. Representative images of three independent experiments are shown. Scale bar = 1000 μm. (**K**,**L**) Fluorescence microscopy images of SPP1 expression in EECs and curves of fluorescence intensity. Representative images of three independent experiments are shown. Scale bar = 100 μm. (**M**) The cell adhesion molecules were measured by real-time quantitative PCR. The data are presented as the means ± SEM of three independent experiments. * Significant difference (*p* < 0.05) compared with other groups; ** Significant difference (*p* < 0.01) compared with other groups.

Given that CRIM1 was robustly stimulated by P_4_, E_2_ and IFN-τ treatment, we were curious whether the loss of CRIM1 affected phenotypic changes of endometrial receptivity. From the data in Figure 3A, it is apparent that the knockdown of CRIM1 inhibited EEC cell proliferation. Meanwhile, the EdU proliferation assay showed similar results to the CCK-8 assay (Figure 3B). The cell cycle profile was detected using flow cytometry by PI staining and analyzed using flowjo software [41]. Figure 3C shows that CRIM1 knockdown disturbed the G1-S transition of cell cycle progression, and this cellular event may account for increased EEC proliferation. Previous studies reported that cyclin-dependent kinase 4 (CDK4) is important for the G1 to S transition during the cell cycle via phosphorylation at T172 [42,43]; we speculated that CDK4 may be involved in CRIM1-mediated cell cycle regulation. As shown in Figure 3D, the expression of the T172-phosphorylated form of CDK4 was decreased in the shCRIM1 group compared with the shN group.

Previous studies demonstrated that the endometrium undergoes violent morphological remodeling to allow embryo attachment. The notable feature is the smooth cell surface without microvilli coverage [44]. To determine the receptivity defects in shCRIM1 EECs, we investigated the ultrastructural changes of the cell surfaces using SEM. From Figure 3E, we can see that microvilli structures could be seen on the surface of shCRIM1 EECs. We further compared the expression of endometrial receptivity markers between shN and shCRIM1 EECs under P_4_, E_2_ and IFN-τ treatments at different time points. Western blot analysis showed that CRIM1 knockdown significantly decreased the protein levels of HOXA10, HOXA11 and ITGB1 compared with that in the shN EECs (Figure 3F). During early pregnancy, the PGs secreted by the endometrium are very important for maintaining the function of the corpus luteum and sustaining pregnancy [45]. We therefore analyzed levels of PGE_2_ and PGF_2α_ secreted by EECs. The results showed that the amount of PGF_2α_ was higher in culture supernatants of shCRIM1 groups than shN groups, but differences between PGE_2_ levels were not statistically significant (Figure 3G). This result prompted us to measure the expression of genes involved in PGs secretion. As expected, knockdown of CRIM1 significantly upregulated the expression levels of PGFS, the dominant rate-limiting enzymes that synthesize PGF_2α_ (Figure 3H). To verify whether knockdown of CRIM1 caused damage to adhesion capacity, spheroid co-culture assays were performed in this study. We found that the number of adhering GTCs spheroids (red) to shCRIM1 EECs (green) was lower than shN EECs (Figure 3I,J). To assess cell adhesion molecules, the fluorescence intensity of SPP1, a key cell adhesion molecule, was observed by immunofluorescence staining. Figure 3K,L show that SPP1 appeared in the cytoplasm and that the fluorescence intensity of SPP1 in the shCRIM1 group was significantly lower than that in the control group. The qPCR results also showed that the expression of integrin genes (ITGB3 and ITGB5) was decreased in the shCRIM1 group compared with the control group (Figure 3M). Together, these data suggest that CRIM1 is a critical regulatory factor in endometrial receptivity, affecting cell proliferation, PG secretion and cell adhesion by regulating downstream gene levels.

### 2.4. Overexpression of ATG7 Reversed Receptivity Defects Phenotype

**Figure 4:**
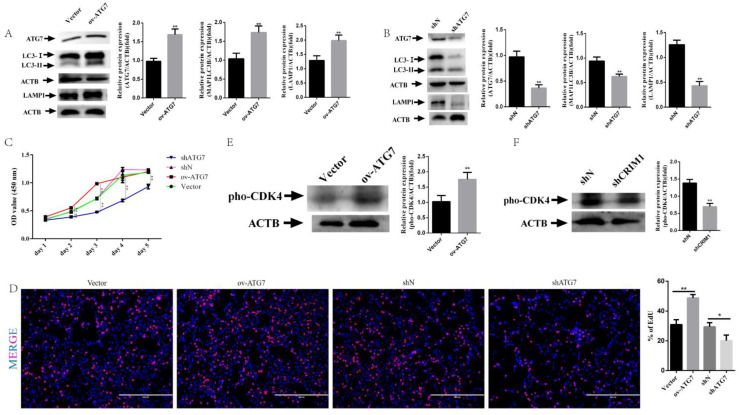
Effect of ATG7 on cell proliferation in shCRIM1 EECs. (**A**,**B**) The shCRIM1 EECs were infected with an empty vector lentivirus (Vector), ATG7 overexpression (ov-ATG7), shN or shATG7 lentivirus for 48 h under P_4_, E_2_ and IFN-τ treatment. Total proteins from the Vector, ov-ATG7, shN and shATG7 groups were subjected to Western blotting. (**C**) Growth curves of the Vector, ov-ATG7, shN and shATG7 groups were determined by the CCK-8 method. The solid line represents the significance between Vector and ov-ATG7 groups; the dotted line represents the significance between shN and shATG7 groups. (**D**) The Vector, ov-ATG7, shN and shATG7 groups were collected to measure DNA synthesis by EdU defect DNA synthesis by EdU proliferation assay. (**E**) Western blot analysis of pho-CDK4 expression in the Vector, ov-ATG7, shN and shATG7 groups. The data are presented as the means ± SEM of three independent experiments. * Significant difference (*p* < 0.05) compared with other groups; ** Significant difference (*p* < 0.01) compared with other groups.

To investigate the necessity of autophagy in CRIM1-regulated endometrial receptivity, we altered autophagy flux by overexpressing ATG7 (ov-ATG7) or silencing ATG7 (shATG7). We infected shCRIM1 EECs with an empty vector lentivirus (Vector), ATG7 overexpression, shN or shATG7 lentivirus. As shown in Figure 4A, the overexpression of ATG7 significantly increased the level of ATG7 and upregulated the expression level of LC3-II and LAMP1. In contrast, knockdown of ATG7 significantly downregulated the ATG7 level and inhibited LC3-II accumulation and LAMP1 expression (Figure 4B). First, we performed a CCK8 assay to measure the effect of overexpressing or silencing ATG7 on the proliferation of shCRIM1 EECs. The results showed that ATG7 overexpression markedly increased cell proliferation in shCRIM1 EECs compared to that in vector control cells, while the inhibition of ATG7 reduced shCRIM1 proliferation (Figure 4C). These results were further confirmed by EdU staining; the results of EdU assays were consistent with the observation of the CCK-8 (Figure 4D). To further validate the above results, we assessed pho-CDK4 by Western blotting. Figure 4E shows that the protein expression of pho-CDK4 was reverted by ATG7 overexpression, whereas ATG7 interference repressed pho-CDK4 expression.

**Figure 5:**
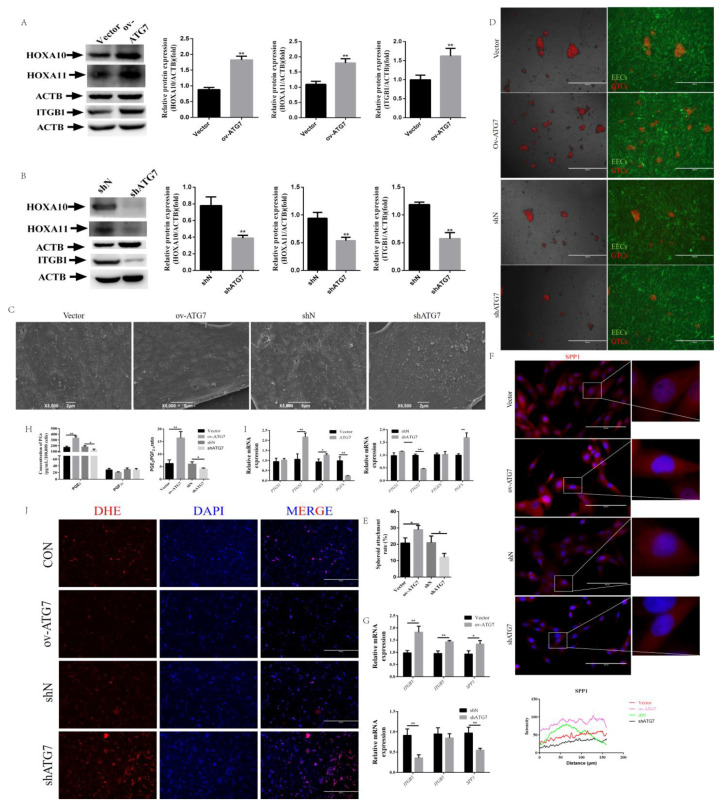
Effect of ATG7 on endometrial receptivity in shCRIM1 EECs. (**A**,**B**) The shCRIM1 EECs were infected with the Vector, ov-ATG7, shN or shATG7 lentivirus for 48 h under P_4_, E_2_ and IFN-τ treatment. Total proteins from the Vector, ov-ATG7, shN and shATG7 groups were subjected to Western blotting. (**C**) The Vector, ov-ATG7, shN and shATG7 groups were treated with P_4_, E_2_ and IFN-τ for 12 h. The EECs were fixed and processed for SEM analysis. (**D**,**E**) GTC spheroids were prepared and the spheroid attachment rate was measured. Representative images of three independent experiments are shown. Scale bar = 1000 μm. (**F**) Fluorescence microscopy images of SPP1 expression in EECs and curves of fluorescence intensity. Representative images of three independent experiments are shown. Scale bar = 100 μm. (**G**) The cell adhesion molecules were measured by real-time quantitative PCR. (**H**) The secretion levels of PGE_2_ and PGF_2α_ were measured in EECs using an ELISA kit. (**I**) The rate-limiting enzymes of synthesized PGs were measured by real-time quantitative PCR. (**J**) The ROS measurement by dihydroethidium probe. The data are presented as the means ± SEM of three independent experiments. * Significant difference (*p* < 0.05) compared with other groups; ** Significant difference (*p* < 0.01) compared with other groups.

Moreover, the expression of endometrial receptivity markers was upregulated in the ATG7 overexpression group compared to the vector group (Figure 5A). However, there was no reversal on the level of marker proteins in shATG7 group and even a decrease in the expression of HOXA10, HOXA11 and ITGB1 (Figure 5B). In further confirmation of these results, we observed changes of cell surface microvilli. The SEM results showed that the microvilli of the ov-ATG7 group were decreased on the cell surface as compared with the vector group, but numerous microvilli of varying lengths were found on the cell surface of the shATG7 group (Figure 5C). We next aimed to determine whether alterations in ATG7 expression levels would affect the cell adhesion of shCRIM1 EECs. The number of adherent GTC spheroids of the ov-ATG7 group was higher than the vector group, but ATG7 interference inhibited the adhesion of GTC spheroids after spheroid placement on the EEC monolayer (Figure 5D,E). Moreover, we also found that the distribution of SPP1 was promoted by the overexpression of ATG7. In contrast, silencing ATG7 decreased the fluorescence intensities of SPP1 (Figure 5F). For integrin genes, we found that ITGB3 and ITGB5 mRNA levels showed similar expression trends with SPP1 results in the ov-ATG7 group (Figure 5G). To further investigate the phenotype of receptivity, we tested their PG secretion after overexpression or interference with ATG7. As shown in Figure 5H, ATG7 overexpression led to increased PGE_2_ secretion and inhibited the secretion of PGF_2α_, although the difference was not significant. The secretion of PGE_2_ was decreased by silencing ATG7 (Figure 5H). It is worthy of note that ATG7 overexpression treatment for shCRIM1 EECs reversed the PGE_2_ to PGF_2α_ ratio; however, the interference of ATG7 decreased the PGE_2_ to PGF_2α_ ratio (Figure 5H). We next measured the dominant rate-limiting enzymes of the synthesis of PGs. We found that the expression of PGFS was induced by shCRIM1 lentivirus, and its induction was inhibited by ATG7 overexpression (Figure 5I). Instead, the knockdown of ATG7 exhibited significantly higher PGFS expression than the shN group (Figure 5I).

Activating autophagy flux at early stages with an overexpression of ATG7 results in divergent receptivity phenotype outcomes compared to an autophagic flux block with ATG7 interference. We aimed to determine whether knockdown of ATG7 results in a disconnect between the endometrial receptivity-related genes and bioenergetic responses. Previous studies have demonstrated that knockdown of ATG7-dependent autophagy increased ROS production [46]. We speculated that knockdown of ATG7 disturbed the sequestration and degradation of cytoplasmic material, such as the impaired mitochondria. Therefore, we sought to measure whether ROS production was different from that of cells treated with ov-ATG7 or shATG7 lentivirus. ROS level was evaluated by dihydroethidium (DHE) staining. As shown in Figure 5J, ATG7 overexpression decreased the production of ROS, while interference of ATG7 promoted ROS production.

### 2.5. miR-143-5p Targeted the CRIM1 3′UTR to Regulate Endometrial Receptivity

**Figure 6:**
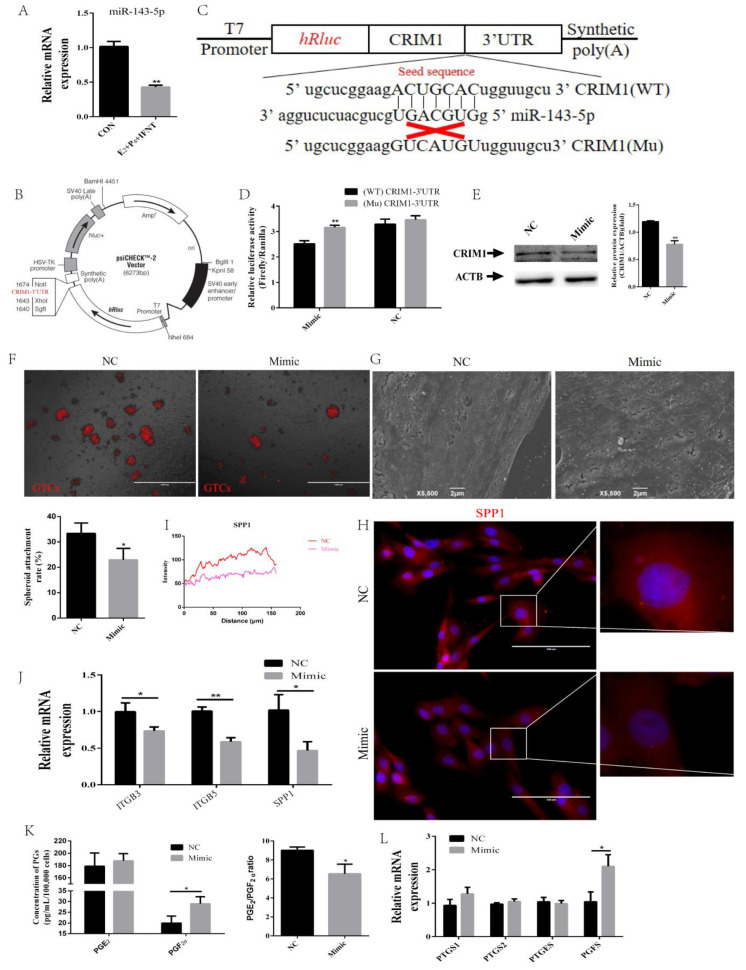
miR-143-5p targeted the CRIM1 3′UTR to regulate endometrial receptivity. (**A**) The effect of P_4_, E_2_ and IFN-τ on the expression levels of miR-143-5p. (**B**) The psiCHECK^TM^-2 vector map and the insertion site of CRIM1-3′UTR are marked in red. (**C**) Schematic diagram illustrating the design of luciferase reporters with the WT-CRIM1 3′ UTR (WT-CRIM1) or the site-directed mutant CRIM1 3′ UTR (Mu-CRIM1). The nucleotides in red represent the “seed sequence” of miR-143-5p. (**D**) The luciferase activity was measured by the dual-luciferase reporter assay system. (**E**) The protein level of CRIM1 was measured by Western blot. (**F**) GTC spheroids were prepared and the spheroid attachment rate was measured. Representative images of three independent experiments are shown. Scale bar = 1000 μm. (**G**) The NC and mimic groups were treated with P_4_, E_2_ and IFN-τ for 12 h. The EECs were fixed and processed for SEM analysis. (**H**,**I**) Fluorescence microscopy images of SPP1 expression in EECs and curves of fluorescence intensity. Representative images of three independent experiments are shown. Scale bar = 100 μm. (**J**) The cell adhesion molecules were measured by real-time quantitative PCR. (**K**) The secretion levels of PGE_2_ and PGF_2α_ were measured in EECs using an ELISA kit. (**L**) The rate-limiting enzymes of synthesized PGs were measured by real-time quantitative PCR. The data are presented as the means ± SEM of three independent experiments. * Significant difference (*p* < 0.05) compared with other groups; ** Significant difference (*p* < 0.01) compared with other groups.

Previous studies have found that the expression of miR-143-5p was obviously decreased to a greater extent in the RE than PE in goat [36]. Through bioinformatics analysis, CRIM1 was found to be a potential target gene of miR-143-5p. As shown in Figure 6A, we found that miR-143-5p was significantly downregulated in the P_4_+E_2_+IFN-τ group compared to the control group. To verify whether CRIM1 is a direct target of miR-143-5p, a 3′-UTR fragment containing the wild-type (WT) or mutant (Mu) miR-143-5p-binding sequences was cloned into psiCHECK^TM^-2 reporter (Figure 6B,C). A dual-luciferase assay showed luciferase activity was significantly decreased with wild-type plasmids but not affected by the mutant plasmids (Figure 6D). Meanwhile, the expression of CRIM1 decreased in EECs after transfection with miR-143-5p mimics (Figure 6E). These results suggested that CRIM1 was a target of miR-143-5p.

To explore the role of miR-143-5p in regulating endometrial receptivity, EECs were transfected with miR-143-5p mimics and NC. In Figure 6F, we observed that the number of attachment GTC spheroids was decreased to a greater extent in the mimics group than the NC group. SEM analysis showed the number of surface microvilli was increased in the mimics group compared to the NC group (Figure 6G). After the transfection of mimics in EECs, the fluorescence intensity of SPP1 was weakened at the cytoplasm (Figure 6H,I). Additionally, the ITGB3 and ITGB5 mRNA levels were lower in the group treated with miR-143-5p mimics than in the group treated with NC in EECs (Figure 6J). Finally, we detected the effect of miR-143-5p mimics on the secretion of PGs. The ELISA results showed that mimics markedly increased the production of PGF_2α_ (Figure 6K). We also found that the mRNA expression of PGFS was upregulated in the mimics group (Figure 6L).

## 3. Discussion

**Figure 7:**
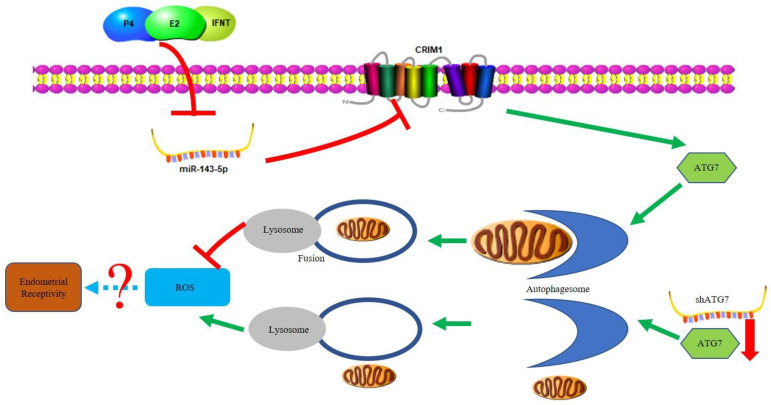
Proposed network of miR-143-5p–CRIM1 autophagy in regulating endometrial receptivity. Our results suggest that hormones and IFN-τ promote CRIM1 expression by inhibiting the level of miR-143-5p, while CRIM1 regulates endometrial receptivity phenotypes through autophagy activation to reduce the production of ROS.

When establishing pregnancy, a critical factor is the ability of the endometrium to accept the embryo, that is, endometrial receptivity. Although it is unclear precisely how to regulate endometrial receptivity in ruminants, there is a large amount of evidence that altered protein levels in EECs during early pregnancy promote embryo implantation. Previous studies have reported that CRIM1 influences cell polarity, proliferation, adhesion and angiogenesis in different tissues and cells [16,17,18]. Zhang et al. reported that CRIM1 was one of the highly expressed genes in the receptive endometrium and showed significant differences between the receptive endometrium and pre-receptive endometrium [15]. Here, we identified a new mechanism by which CRIM1-regulated goat endometrial receptivity was dependent on autophagy. The activation of autophagy flux restored endometrial receptivity defects caused by CRIM1 deficiency via overexpressing ATG7 instead of ATG7 interference. Moreover, we demonstrated that miR-143-5p targeted CRIM1 3′UTR and that treatment with miR-143-5p mimics results in the impairment of endometrial receptivity phenotypes. Together, these results suggest that hormones and IFN-τ promote CRIM1 expression by inhibiting the level of miR-143-5p, while CRIM1 regulates endometrial receptivity phenotypes through autophagy activation to reduce the production of ROS (Figure 7).

CRIM1 is highly expressed in multiple type of cells and tissues, including the reproductive tract [47]. Previous studies have demonstrated that CRIM1 is necessary for epithelial cell polarity and proliferation during lens development [18]. Zhang et al. reported that CRIM1 can bind to integrin to regulate cell adhesive interactions [16]. In addition to cell proliferation, cell adhesion and polarity also occur in the endometrium during early pregnancy. We found in our culture model that CRIM1 was increased following treatment with P_4_, E_2_ and IFN-τ. To elucidate the roles of CRIM1 on endometrial receptivity, we constructed the shCRIM1 lentiviral vectors by using the third-generation lentiviral packaging system. Previous studies have suggested that the deletion of CRIM1 leads to a slower proliferation rate [48]. Our results were consistent with prior studies. Our current study showed that CRIM1 depletion inhibited EEC proliferation and prolonged the G1 to S transition. It is currently unclear how CRIM1 influences cell proliferation, but it apparently plays an important role in cell proliferation by regulating integrin signaling [16]. The related molecular mechanism is worthy of further exploration.

During early pregnancy, endometrial development resulting in endometrial receptivity requires ultrastructural changes and the expression of a variety of molecules to facilitate the adhesion of the embryo to the uterine endometrium [49]. The ultrastructural changes include a decrease in the number of microvilli covering the epithelial cells of the endometrium [44]. We observed that the loss of CRIM1 impaired the ability to perform microvilli retraction. We also measured cell adhesion by performing a spheroid co-culture assay, which is a widely used method to detect cell viability in vitro. Our results showed that the knockdown of CRIM1 decreased the percentage of the attached GTC spherioids to EECs. We aimed to determine whether the lack of cell adhesion activity of shCRIM1 EECs was due to the inhibition of cell adhesion molecules. Recent studies have demonstrated that the functions of HOXA10 and HOXA11 were to regulate the expression of integrin and implantation efficiency [44]. D’Occhio et al. reported that robust integerin expression in the endometrium facilitates embryo attachment [50]. Moreover, the function of SPP1 binding alphavbeta3 and alpha5beta1 integrins to induce cell adhesion has been demonstrated [51]. It is clear that CRIM1 physically interacts with integrins; our own observations suggest that HOXA10, HOXA11, SPP1 and the integrin levels were reduced on CRIM1 knockdown in EECs. These findings led us to speculate that CRIM1 functions as a cell adhesion regulator, which may affect HOXA10, HOXA11 and SPP1 expression via a direct interaction with integrins. More research needs to be conducted regarding this speculation. During early pregnancy, the synthesis of PGs by the endometrium is critical for maintaining the function of the corpus luteum in ruminants [52]. The general function of endometrial PGE_2_ is associated with the luteotrophic pathway and maternal recognition of pregnancy, while endometrial PGF_2α_, unlike PGE_2_, has properties that activate luteolytic actions [52]. The optimal PGE_2_ to PGF_2α_ ratio has been considered to be a critical event in the establishment of endometrial receptivity. The endometrium PGs synthesis from arachidonic acid (AA) is dependent on the expression of multiple enzymes, including cyclooxygenase 1 (PTGS1) and cyclooxygenase 2 (PTGS2). The downstream prostaglandin synthases catalyze PGH_2_ is converted into PGF_2α_ and PGE_2_ by prostaglandin F synthase (PGFS) and prostaglandin E synthase (PTGES), respectively [53]. In this study, the inhibition of CRIM1 downregulated the PGE_2_ to PGF_2α_ ratio and increased the PGFS level. Previous studies reported that CRIM1 can act as a BMP antagonist by binding with BMPs, thus inhibiting their maturation and secretion [16]. Zhang et al. demonstrated that BMP4 directly regulates the expression of the key enzyme for PG synthesis [54]. The hypothesis that CRIM1 regulates PG synthesis by binding with BMPs will need to be confirmed by further studies.

Autophagy is hormonally regulated in the endometrium and plays essential roles in physiological and pathological processes [55]. Additionally, autophagy is involved in energetic metabolism via recycling metabolites to promote the survival of EECs and trophoblasts under low oxygen and low nutrient conditions of the maternal–fetal interface [56]. Toschi et al. reported that the autophagy pathway is required for vasculogensis and placental development during early pregnancy in sheep [57]. Consistent with the literature, our results found that autophagic flux was activated in our EEC culture model by the pregnancy hormones and IFN-τ treatment. Our results found that CRIM1 negatively regulated the autophagic flux in EECs under P_4_, E_2_ and IFN-τ treatments. Previous studies have found that CRIM1 interacts with BMP4/7 at the cell surface and inhibits BMP secretion, and BMP signaling regulates autophagy by mTORC1 activity [58,59]. Based on current knowledge, it seems reasonable to propose that CRIM1 regulates autophagy by targeting BMP signaling in EECs, but our results failed to reveal the detailed molecular mechanism; more research is needed on this topic.

We next aimed to determine whether CRIM1-mediated autophagy regulated endometrial receptivity. To evaluate the requirement of autophagy in CRIM1-regulated endometrial receptivity, ATG7 was targeted for activation or inhibition. Current evidence suggests that ATG7 interacts with ATG3 and ATG5-12 to facilitate the phagophore elongation and closure [60]. Aoki et al. found that autophagy deficency induced the failure of vascular remodeling in the placenta-specific ATG7 conditional knockout mice [61]. However, whether ATG7 participates in the establishment of endometrial receptivity in ruminants is currently unknown but is worth further investigation. We found that the receptivity defect phenotype due to CRIM1 interference was restored by ATG7 overexpression in EECs, while the loss of ATG7 further impaired cell proliferation, cell adhesion and the PGE_2_ to PGF_2α_ ratio. Moreover, our results showed that changing the expression of ATG7 affected ROS production. ROS-mediated autophagy has been reported in various physiological and pathophysiological processes, where autophagy inhibition at the early stages of autophagic flux induced ROS production by mitochondria accumulation [62]. Excess ROS had a direct negative effect on the endometrium, which normally functions to support embryo implantation [63]. However, the relationship between ROS and CRIM1-mediated endometrial receptivity remains to be further studied.

The miR-143-5p was expressed to a lower level in the receptive endometrium of goats, with higher expression in the pre-receptive endometrium [36]. Recent studies found that miR-143 plays an important role in glucose metabolism and mitochondrial function in the placenta [64]. Moreover, uterine miR-143 has been shown to be involved in cell proliferation, migration and invasion by binding to 3′UTR of leukemia inhibitory factor receptor in human endometrial stromal cells during early pregnancy [37]. Yuan et al. demonstrated that miR-143 was regulated by hormones in human endometrial epithelial cells and inhibited the proliferation of human endometrial cancer cells [65]. Moreover, E_2_ can downregulate the miR-143 expression in vivo and in vitro [66]. Through bioinformatics analysis, we found that CRIM1 was identified as a possible target gene of miR-143-5p. Our results demonstrated that miR-143-5p downregulated CRIM1 at the protein level and hindered the function of EECs by regulating cell adhesion and PG secretion. According to the above studies, we speculate that hormones and IFN-τ increase the CRIM1 expression by inhibiting miR-143-5p. However, a single mRNA may be regulated by multiple miRNAs; there may be other miRNAs that regulate the CRIM1, and whether hormones and IFN-τ have a direct association with CRIM1 remains unknown. Further studies are required to verify more detailed molecular mechanisms.

This study revealed that CRIM1 was a critical factor in regulating endometrial receptivity, providing important information to understand the function of CRIM1 during early pregnancy in goat. Dynamic changes in the endometrial epithelium occur during the establishment of endometrial receptivity that are primarily regulated in ruminants under P_4_, E_2_ and IFN-τ treatment. The results showed that P_4_, E_2_ and IFN-τ treatment upregulated the CRIM1 expression and activated autophagy. We confirmed that CRIM1 interference hindered cell proliferation, cell adhesion and PG secretion under P_4_, E_2_ and IFN-τ treatment. The results strongly supported the idea that CRIM1, as the downstream target of miR-143-5p, affects the ATG7-dependence of EECs, and it may mediate the regulation of endometrial receptivity through this mechanism. Moreover, the formation of ROS may be a crucial signaling molecule for the development of endometrial receptivity, warranting further investigation. Understanding the function of miR-143-5p–CRIM1 autophagy in endometrial receptivity is essential for searching for new targets for diagnosis and treatment of endometrial receptivity defects and increasing artificial reproduction success rates in ruminants.

## 4. Materials and Methods

### 4.1. Cell Culture and Drug Treatment

The human telomerase reverse transcriptase (hTERT) was employed to induce immortalized goat endometrial epithelial cells (EECs) and goat trophoblast cells (GTCs) [67,68,69]. The EECs and GTCs were seeded in six-well plates with DMEM/F-12 medium, supplemented with 10% fetal bovine serum (FBS, AusgeneX, Loganholme, QLD, Australia).

According to our previous description, the EECs were cultured in fresh DMEM/F-12 plus 0.1% bovine serum albumin (BSA) when cell density reached 70–80% confluence [70]. Then, P_4_ (10^−7^ M, Sigma, St. Louis, MO, USA) and E_2_ (10^−9^ M, Sigma, St. Louis, MO, USA) were added to the medium. After hormone treatment for 12 h, the EECs were treated with 20 ng/mL IFN-τ (Sangon Biotech Co., Ltd., Shanghai, China) for 4 h, 8 h or 12 h. In the presence of CQ (Sigma, St. Louis, MO, USA) groups, 10 μM CQ was added to EECs before adding IFN-τ.

### 4.2. Cell Transfection and Fluorescence Measurement

To generate FUGW-GFP-LC3-RFP, recombinant lentiviral vectors encoding the GFP-LC3-RFP were constructed as previously described [39]. Goat MAP1LC3B cDNA (GenBank accession number: XM_018061829.1) was used. Virus packaging and cell transfection were performed as previously reported [71]. After P_4_, E_2_ and IFN-τ treatment, cells were fixed with 4% PFA for 15 min and washed with PBS. The cells were observed by a fluorescence microscope (Nikon Inc., Melville, NY, USA) and measured for GFP and RFP fluorescence by ImageJ (Rockville, MD, USA).

Recombinant lentiviral vectors encoding the CRIM1 shRNA (shCRIM1), the ATG7 shRNA (shATG7) and negative control short hairpin RNA (shN) were constructed as previously described [70]. The sequences of shCRIM1, shATG7 and shN are shown in Supplementary Table S1. Virus packaging and cell transfection were performed as previously reported [71].

The goat ATG7 gene was amplified and cloned into the pCD513B vector to synthetize pCD513B-ATG7. Virus packaging and cell transfection were performed as previously reported [71].

### 4.3. Spheroid Co-Culture Assay

Single GTC suspensions were labeled with CellTracker CM-DiI (1 μM, Yeasen Biotech Co., Ltd., Shanghai, China) and seeded at 2500 cells per well in non-adherent round-bottomed 96-well plates to encourage spheroid development. The GTC spheroids (approximately 50 spheroids per well) were delivered onto EECs, which were treated with hormones and IFN-τ as previously described [70]. The co-culture system was shaken for 10 min at 110 rpm after 1 h of incubation. The number of GTC spheroids remaining in wells were counted, and the attachment rate was expressed as a percentage of seeded spheroids.

### 4.4. RNA Extraction and Real-Time Quantitative PCR

Total RNA was extracted from cells by TRIzol reagent (TaKaRa Bio, Inc., Dalian, China) and cDNA was synthesized using a ABScript II RT Master Mix for qPCR (ABclonal Biotechnology, Wuhan, China). The primer sequences are shown in Supplementary Table S2. Real-time quantitative PCR was carried out using ABclonal 2X Universal SYBR Green Fast qPCR Mix (ABclonal Biotechnology, China) in Step One Real-Time PCR System (Applied Biosystems, Carlsbad, CA, USA). The 2^−∆∆Ct^ method was employed to estimate the expression. The expression of mRNA was normalized by the *GAPDH* gene and U6 for miR-143-5p.

### 4.5. Western Blot Analysis

After the treatment, EECs were collected and washed with ice-cold PBS and lysed with RIPA buffer (Beijing Solarbio Science & Technology Co., Ltd., Beijing, China). The total protein concentration was measured by the BCA assay (Nanjing Keygen Biotech Co., Ltd.). Thirty micrograms of total protein were loaded into each well of a 12% SDS-PAGE gel, and the proteins were then separated by electrophoresis. Proteins were then transferred to PVDF membranes (Millipore; Bedford, MA, USA). After blocking in Tris-buffered saline containing 0.5% Tween-100 (TBST) with 10% nonfat milk for 2 h, the samples were incubated with anti-CRIM1 (Bioss bs-2034R, diluted 1:1000), anti-LC3 antibody (Sigma L7543, diluted 1:1000), anti-SQSTM1 antibody (CST 8025, diluted 1:1000), anti-phospho-mTOR (Ser2448) (CST 2974, diluted 1:1000), anti-LAMP1 antibody (CST 9091, diluted 1:1000), anti-ATG7 antibody (CST 8558, diluted 1:1000), anti-HOXA10 antibody (ABclonal A8550, diluted 1:1000), anti-HOXA11 antibody (ABclonal A2976, diluted 1:1000), anti-ITGB1 antibody (ABclonal A2217, diluted 1:1000), anti-pho-CDK4^T172^ antibody (ABclonal AP0593, diluted 1:1000) and anti-ACTB antibody (Proteintech Group, Inc., Wuhan, China, diluted 1:2000) overnight at 4 °C. Subsequently, the membranes were incubated for 1 h with HRP-labeled secondary antibody at room temperature. Finally, the protein bands were visualized using the Image-Pro plus 6.0 software (Media Cybernetics, Inc., Silver Spring, MD, USA) and measured with Quantity One software (Bio-Rad Laboratories, Hercules, CA, USA).

### 4.6. Immunofluorescent Staining

The method employed for the subsequent steps followed the method described in our previous publication [70]. Briefly, after blocking in 5% BSA in PBS for 1 h, the samples were incubated with primary antibodies at 37 °C for 2 h, including anti-SPP1 (Wanleibio Co., Ltd. Shenyang, China, WL02378, diluted 1:150) and anti-TFEB (ABclonal A7311, diluted 1:200). After being washed three times with PBS and incubated for 1 h at room temperature in a 1:500 dilution mixture of Alexa-labeled donkey anti-rabbit IgG (Invitrogen, Life Technologies) at 37 °C for 2 h, the nuclei were counterstained by DAPI (4,6-diamidino-2-phenylindole), and the cells were observed by a fluorescence microscope (Nikon Inc., Melville, NY, USA).

### 4.7. Prostaglandins Measurement

EECs were plated into 24-well plates (5 × 10^4^ cells/well) and cultured as previously described [72]. After incubating with IFN-τ for 12 h, the culture supernatants were collected, and the concentrations of PGF_2α_ and PGE_2_ were measured by the Goat Prostraglandin F2α ELISA kit (Shanghai Enzyme-Linked Biotechnology Co., Ltd., Shanghai, China) or Goat Prostraglandin E2 ELISA kit (Shanghai Enzyme-Linked Biotechnology Co., Ltd. China). The cells were counted by a cell count plate.

### 4.8. Measurement of Cell Viability

The EECs (5 × 10^3^ cells/well) were seeded in 96-well plates with 10 μL of the Cell Counting Kit-8 (CCK-8, Beyotime, Haimen, Jiangsu, China) for 2 h at 37 °C. The OD value was measured at 450 nm by a Microplate Reader (Bio-Rad 680).

### 4.9. Cell Cycle Analysis

The EECs infected with shN or shCRIM1 were washed with ice-cold PBS and harvested with trypsin. Next, the cells were centrifuged at 2000 rpm for 5 min and fixed in cold 70% ethanol overnight at 4 °C. All samples were then stained using the Cell Cycle Detection Kit (Multisciences (lianke) biotech, Co., Ltd., Shanghai, China) as we have previously reported [72]. Finally, the samples were analyzed by flow cytometry. Each test was performed in triplicate.

### 4.10. EdU Proliferation Assay

EECs were cultured in 24-well plates with 5 × 10^4^ cells/well and then incubated with 10 μM EdU for 6 h. After removing the medium, the EECs were fixed and washed with PBS plus 3% BSA. After permeabilizing, the EECs were stained according to the manufacturer’s instructions. The samples were observed by fluorescence microscope (Nikon Inc., Melville, NY, USA).

### 4.11. Transmission Electron Microscopy (TEM)

TEM analysis was performed following previous studies [73]. Briefly, the cell samples were fixed with glutaraldehyde for 48 h, then dehydrated in a graded ethanol series and embedded. The ultrathin sections were mounted on nickel grids. The samples were stained and visualized with a JEM-1400.

### 4.12. Scanning Electron Microscopy (SEM)

SEM analysis was performed following previous studies [70]. Briefly, the cell samples were fixed with glutaraldehyde for 48 h, then dehydrated in a graded ethanol series and critical-point dried. The cell samples were coated with gold and observed with a JSM-6390LV.

### 4.13. Assessment of Reactive Oxygen Species (ROS) Generation

EECs were cultured in 24-well plates with 5 × 10^4^ cells/well, then incubated with dihydroethidium (5 μM) for 30 min. After removing the medium, the EECs were washed with PBS. Then, the EECs were stained with DAPI according to the manufacturer’s instructions. The samples were observed by a fluorescence microscope (Nikon Inc., Melville, NY, USA).

### 4.14. 3′-UTR Constructs/Luciferase Assay

The method employed for the steps followed the method described in a previous publication [35]. Briefly, luciferase reporter constructs were generated with psiCHECKTM-2 vector containing insert target sites in the 3′-UTR of CRIM1 mRNA. The EECs were plated into 24-well plates (5 × 10^4^ cells/well) and co-transfected with wild-type (psiCHECK-CRIM1-WT) or mutated (psiCHECK-CRIM1-MU) and miR-143 mimics or a negative control (NC). At 24 h post-transfection, renilla and firefly luciferase activities were measured by the Dual-Glo luciferase assay system (Yeasen Biotech Co., Ltd., Shanghai, China).

### 4.15. Statistical Analysis

Unless otherwise specified, all data are expressed as the mean ± SD. A one-way ANOVA was performed, followed by Fisher’s least significant difference (LSD) or Student’s *t*-test. Statistical differences were considered significant when the *p* value was less than 0.05.

## Data Availability

Data are available upon reasonable request by email to corresponding author.

## Supplementary Materials

The following are available online at https://www.mdpi.com/article/10.3390/ijms22105323/s1, Supplementary Table S1: Short hairpin interfering RNA (shRNA) inserts. Supplementary Table S2: Primer pairs used for real-time quantitative PCR.
