# Supplementary material for: Essential Role of CRIM1 on Endometrial Receptivity in Goat
Source: Int J Mol Sci. 2021 May 18;22(10):5323. doi: 10.3390/ijms22105323 (PMC8158520; doi:10.3390/ijms22105323)
Supplement: Supplementary file 1 [file ijms-22-05323-s001.zip › ijms-1168438-supplementary/Supplementary Table1.pdf]

Supplementary Table. S1**.**

Short hairpin interfering RNA (shRNA) inserts.

shRNA	Sequence (loop in bold letters) (5' to 3')
shCRIM1-1	GATCCGCCCTTCTGTATCCTGTGAAA**CTCGAG**TTTCACAGGATACAGAAGGGCTTTTTG
	AATTCAAAAAGCCCTTCTGTATCCTGTGAAA**CTCGAG**TTTCACAGGATACAGAAGGGCG
shCRIM1-2	GATCCGGTATCGAACACCAACTAAGC**CTCGAG**GCTTAGTTGGTGTTCGATACCTTTTTG
	AATTCAAAAAGGTATCGAACACCAACTAAGC**CTCGAG**GCTTAGTTGGTGTTCGATACCG
shCRIM1-3	GATCCGGGAAGATGACTGCACGTTCT**CTCGAG**AGAACGTGCAGTCATCTTCCCTTTTTG
	AATTCAAAAAGGGAAGATGACTGCACGTTCT**CTCGAG**AGAACGTGCAGTCATCTTCCCG
shATG7	GATCCGCTACAAACTTGGCTGCTACT**CTCGAG**AGTAGCAGCCAAGTTTGTAGCTTTTTG
	AATTCAAAAAGCTACAAACTTGGCTGCTACT**CTCGAG**AGTAGCAGCCAAGTTTGTAGCG
shN	GATCCTTCTCCGAACGTGTCACGT**TTCAAGAGA**ACGTGACACGTTCGGAGAATTTTTTG
	AATTCAAAAAATTCTCCGAACGTGTCACGT**TCTCTTGAA**ACGTGACACGTTCGGAGAAG
