# Supplementary material for: Essential Role of CRIM1 on Endometrial Receptivity in Goat
Source: Int J Mol Sci. 2021 May 18;22(10):5323. doi: 10.3390/ijms22105323 (PMC8158520; doi:10.3390/ijms22105323)
Supplement: Supplementary file 1 [file ijms-22-05323-s001.zip › ijms-1168438-supplementary/Supplementary Table2.pdf]

Supplementary Table S2. Primer pairs used for Real time quantitative PCR

Gene	Sequences(5´→3´)	References or GenBank accession number
ITGB3	Forward: acggtgagcttcagcattga	XM_018047091.1
	Reverse: acaccccacactcaaaggtc	
ITGB5	Forward: cccacgagaaggctacttgg	XM_018047092.1
	Reverse: ttcaacaggcgtctcgatcc	
SPP1	Forward: tgagaattgcagtgatttgc	XM_005680968.3
	Reverse: tgagatgggtcaggctttag	
PTGS1	Forward: tcacagtgcgttccaaccttatc	XM_005687044.3
	Reverse: acggagggcagaatgcgagta	
PTGS2	Forward: gagtgtaggattcgaccagtat	XM_018060731.1
	Reverse: ccttgaagtgggtaagtatgtag	
PTGES	Rorward: catcaaaatgtacgcggtggc	XM_018055956.1
	Reverse: gtcctcggggttggcaaaagc	
PGFS	Forward: tggaggacccagttctttgtg	XM_004014323.3
	Reverse: tacctgatagcgaagggcaac	
GAPDH	Forward: gatggtgaaggtcggagtgaac	XM_005680968.3
	Reverse: gtcattgatggcgacgatgt	
miR-143-5p-loop	gtcgtatccagtgcagggtccgaggtattcgcactggatacgacgagcta	/
miR-143-5p	Forward: cgcgtgagatgaagcactg	/
	Reverse: agtgcagggtccgaggtatt	
U6	Forward: ctcgcttcggcagcaca	/
	Reverse: aacgcttcacgaatttgcgt	
